# Profound Hypokalaemia and Functional Weakness Secondary to Gastric Outlet Obstruction: A Rare Case

**DOI:** 10.7759/cureus.94015

**Published:** 2025-10-07

**Authors:** Abimbola Aduroja, Kwerinuchi T Kejeh

**Affiliations:** 1 Internal Medicine, Health Education England, Newcastle Upon Tyne, GBR; 2 Internal Medicine, North Tees Hospital, Stockton-on-Tees, GBR

**Keywords:** gastric outlet obstruction, hypokalemic quadriparesis, malnutrition, pyloric stenosis, recurrent gastric outlet obstruction, severe hypokalaemia

## Abstract

We present the case of a 44-year-old male patient with a known history of pyloric stricture secondary to peptic ulcer disease who was admitted with quadriparesis, vomiting, and chest pain. He had a background of multiple endoscopic dilatations for gastric outlet obstruction and was severely malnourished at presentation. Clinical findings included markedly low serum potassium, phosphate, and magnesium, elevated creatine kinase, and ECG changes suggestive of myocardial strain. High-sensitivity troponin was also elevated, prompting evaluation for acute coronary syndrome and aortic dissection. Imaging ruled out structural cardiovascular pathology, and a diagnosis of type 2 myocardial infarction was made, likely secondary to electrolyte imbalance and volume depletion.

Despite correction of electrolyte abnormalities, the patient’s neuromuscular weakness persisted, prompting neurologic evaluation and spinal imaging to exclude Guillain-Barré Syndrome and compressive pathology. MRI spine was unremarkable. Over the course of his intensive care admission, the patient received central electrolyte replacement, cautious nutritional rehabilitation, and multidisciplinary input from neurology, cardiology, gastroenterology, and dietetics. As electrolyte levels improved, so did his neurological function, and he regained significant muscle strength.

He was subsequently transferred to the gastroenterology ward, where further endoscopic assessment confirmed ongoing pyloric narrowing. Given his history of multiple unsuccessful dilatations and persistent symptoms, he was referred to surgical services for consideration of a definitive gastrojejunostomy.

This case highlights the importance of considering gastrointestinal causes of profound electrolyte disturbances and the potential for these metabolic abnormalities to mimic primary neuromuscular or cardiac pathologies. It also underscores the critical role of multidisciplinary collaboration in managing complex cases involving metabolic, nutritional, and functional decline. Early recognition and a systematic approach to diagnosis and treatment can prevent complications, improve outcomes, and avoid unnecessary interventions.

## Introduction

Electrolyte disturbances such as hypokalaemia and hypophosphataemia can result in profound neuromuscular and cardiovascular complications, particularly in patients with underlying gastrointestinal pathology. Chronic vomiting, as seen in gastric outlet obstruction (GOO), can precipitate severe metabolic derangements and nutritional deficiencies, contributing to significant morbidity if not promptly recognised and managed [1].

Pyloric stricture is a recognised complication of peptic ulcer disease (PUD), particularly in individuals with chronic non-steroidal anti-inflammatory drug (NSAID) use, *Helicobacter pylori* infection, or ongoing mucosal inflammation [2]. Repeated duodenal ulceration can result in the fibrotic narrowing of the pyloric channel, ultimately causing GOO and persistent vomiting [2]. This clinical scenario predisposes patients to profound hypokalaemia, metabolic alkalosis, and refeeding syndrome upon nutritional intervention [3].

This report highlights a rare and clinically complex presentation of functional weakness, type 2 myocardial infarction (T2MI), and profound hypokalaemia in the context of malnutrition from a refractory pyloric stricture. The case underscores the importance of recognising gastrointestinal causes of metabolic decompensation and the need for multidisciplinary care in stabilising and rehabilitating such patients.

## Case presentation

A 44-year-old male patient with a known history of NSAID-induced duodenal ulceration, which led to the development of a refractory pyloric stricture, presented to the Emergency Assessment Unit (EAU) with progressive limb weakness, vomiting, and severe fatigue. He had undergone a total of seven endoscopic dilatations for this stricture, the most recent being in April 2025.

The patient reported a three-week history of declining oral intake and early satiety due to recurrent postprandial vomiting. He described new-onset heaviness and pain in his legs, which later progressed to involve his upper limbs. On the day of admission, he experienced central chest pain radiating to the epigastrium, described as throbbing and non-pleuritic.

Initial evaluation revealed a cachectic man who appeared malnourished. Neurological examination revealed that the patient was alert and orientated. Cranial nerves were intact. Muscle strength was reduced: right wrist dorsiflexion and right elbow flexion were 2/5, with generally reduced power in all limbs: 1/5. Reflexes and sensation were preserved. Plantar reflexes were downgoing.

**Figure 1:**
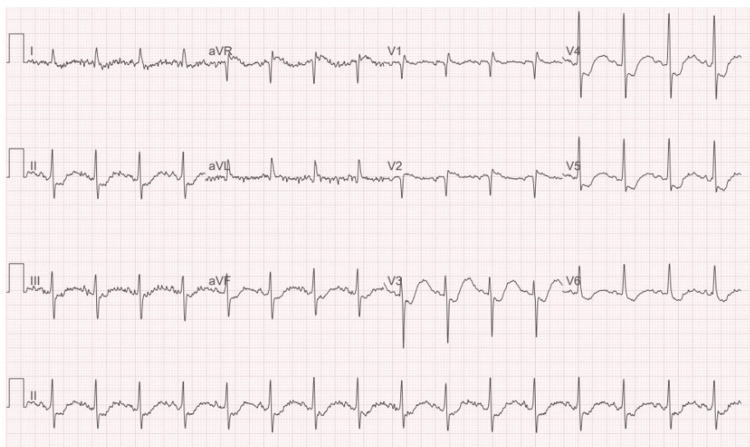
ECG findings at presentation

Given the presence of chest pain, elevated high-sensitivity troponin I levels (135 ng/L), and ECG findings suggestive of posterior infarction (Figure 1), an initial working diagnosis of acute coronary syndrome (ACS) or even aortic dissection was considered. Cardiologist input was urgently sought, and an urgent chest X-ray and blood panel, including D-dimer and serial troponins, were requested. A CT aorta was performed urgently, which ruled out aortic dissection. Subsequent bedside echocardiography did not reveal any regional wall motion abnormalities, and the cardiology team concluded that the findings were most consistent with a T2MI secondary to severe electrolyte imbalance.

Parallel to cardiac evaluation, metabolic investigations revealed a normal thyroid function and vitamin B12 (thyroid-stimulating hormone (TSH): 2.47 mU/L, B12: 241 ng/L), marked hypokalaemia (K+ 1.6 mmol/L), hypophosphataemia (0.68 mmol/L), and elevated creatine kinase (CK) (1,710 → 2,258 U/L), suggestive of rhabdomyolysis. Arterial blood gas (ABG) showed a pH of 7.33, bicarbonate of 15.7 mmol/L, and chloride of 122 mmol/L, confirming a mild metabolic acidosis. These abnormalities, combined with ECG changes suggestive of myocardial strain, prompted urgent transfer to the intensive care unit (ICU) for central electrolyte replacement.

Although electrolyte replacement was initiated and serum levels began to improve, the patient's neuromuscular symptoms remained concerning. The neurology team was contacted for further evaluation. Based on the clinical presentation, they recommended investigations to exclude alternative causes of motor deficits, including Guillain-Barré Syndrome (GBS) and compressive spinal pathology. An MRI spine was arranged to assess for transverse myelitis, compressive lesions, or other cord involvement. The imaging returned normal, ruling out structural spinal cord pathology.

Nutritional assessment revealed a weight of 59 kg and BMI of 18.4, with a 13.5% weight loss from baseline. A MUST score of 2-3 indicated high risk for refeeding syndrome [4]. A cautious nutritional rehabilitation programme was initiated, including Fortijuce, Pabrinex, and oral multivitamins under the supervision of the dietitian.

During his time in the ICU, the patient received central electrolyte replacement therapy under continuous cardiac and neurological monitoring. Serum potassium, phosphate, and magnesium levels were closely observed and corrected. In addition to nutritional optimisation, supportive therapies included intravenous fluids, glucose supplementation, thiamine, and multivitamins to mitigate the risk of refeeding syndrome. The patient was reviewed daily by the ICU, cardiology, neurology, and dietetic teams.

As his electrolyte profile improved, so did his neurological symptoms. The patient developed increased muscle strength in both upper and lower limbs, with noticeable recovery of function and endurance. He began tolerating oral nutrition and showed enhanced strength and mobility. Once stabilised and no longer requiring invasive monitoring or central electrolyte replacement, he was transferred to the gastroenterology ward for ongoing care, including definitive investigation and management of his pyloric stricture.

On the gastroenterology ward, the patient continued to receive oral electrolyte supplementation and nutritional support. Serial laboratory tests confirmed stable biochemical parameters (Table 1). He underwent further endoscopic evaluation, which confirmed pyloric narrowing without active ulceration. Plans were made for repeat endoscopic balloon dilatation. Physiotherapy was commenced to support continued functional recovery, and the patient was reviewed regularly by the nutrition and gastroenterology teams until he was deemed fit for discharge with outpatient follow-up.

**Table 1:** Trend in serum electrolytes and renal function during admission eGFR: estimated glomerular filtration rate

Date	Magnesium (mmol/L)	Phosphate (mmol/L)	Calcium (mmol/L)	Sodium (mmol/L)	Urea (mmol/L)	Creatinine (µmol/L)	eGFR (mL/min/1.73 m²)
Day 1	1.08	0.39	2.34	141	7.1	116	66
Day 2	0.99	0.68	2.3	142	5.5	104	75
Day 3	1.23	1.11	2.06	148	4.2	81	>90
Day 4	0.89	0.78	2.36	143	2.9	87	>90
Day 5	0.79	0.9	2.32	146	2.8	98	80

At outpatient follow-up with the surgical team, the patient was categorised as having failed medical and endoscopic management due to ongoing symptoms and endoscopic evidence of significant pyloric narrowing. In view of this, the surgical team is currently considering definitive surgical intervention in the form of subtotal gastrectomy with Roux-en-Y gastrojejunostomy. Preoperative investigations including CT imaging to delineate anatomy and serum gastrin and gut hormone profiling have been initiated to support surgical planning. A follow-up consultation was arranged to finalise the management plan pending these results.

## Discussion

This case illustrates a diagnostically challenging and clinically significant overlap between gastrointestinal obstruction, profound metabolic disturbances, and reversible neuromuscular dysfunction. While pyloric stricture is a recognised complication of PUD, its acute presentation with quadriparesis and biochemical derangements is rare in the Western world, where widespread *H. pylori* eradication and proton pump inhibitor use have reduced the incidence of such complications [1,2].

Hypokalaemia is well recognised to mimic acute neuromuscular syndromes, and case reports have documented flaccid paralysis due to electrolyte loss from pyloric stenosis [5,6]. However, in most published accounts, muscle weakness improves promptly once potassium is corrected [6]. Our patient’s persistent paralysis despite initial repletion necessitated neurological evaluation and spinal imaging, reflecting the severity of concurrent hypophosphataemia, hypomagnesaemia, and malnutrition. The overlap of these deficiencies likely prolonged neuromuscular dysfunction, contrasting with prior reports where isolated hypokalaemia explained the weakness [3,6]. This case, therefore, underlines that combined electrolyte derangements and refeeding risk can produce a more protracted clinical course, occasionally mimicking GBS both clinically and diagnostically.

In literature, chronic GOO more commonly presents with progressive vomiting, weight loss, and classical hypochloraemic, hypokalaemic metabolic alkalosis [1,7,8]. Reports from Europe and North America describe patients presenting with severe alkalosis, dehydration, and occasionally arrhythmia, with diagnosis often guided by overt gastrointestinal symptoms [7,8]. In contrast, our patient’s initial presentation was dominated by neuromuscular weakness and chest pain with ECG changes, leading to urgent evaluation for GBS, ACS, and aortic dissection. This highlights a key difference: whereas published cases emphasise gastrointestinal clues, our patient’s presentation was multisystemic and initially misleading, complicating the diagnostic trajectory.

The patient’s presentation was also complicated by malnutrition and high refeeding risk. GOO, by limiting nutrient intake and causing chronic vomiting, predisposes patients to micronutrient depletion and muscular catabolism [1,3]. The reintroduction of nutrition without proper planning can unmask electrolyte shifts characteristic of refeeding syndrome, with hypophosphataemia, hypomagnesaemia, and worsening hypokalaemia contributing to cardiac and neurological instability [6]. Timely identification, cautious nutritional repletion, and prophylactic supplementation, as undertaken here, are fundamental to safe recovery.

Cardiac manifestations also distinguished our case. Although ECG abnormalities are well described in hypokalaemia, and hypokalaemia and electrolyte disturbance are well documented to cause a T2MI [5,9], reports of T2MI secondary to electrolyte imbalance in GOO are limited. Our patient’s elevated troponin and chest pain initially prompted an ACS workup, which is rarely emphasised in previous cases. This divergence underscores the potential for metabolic crises to masquerade as primary cardiac emergencies, adding complexity to clinical reasoning.

Treatment approaches also differed. Literature suggests many benign strictures improve with endoscopic dilatation and medical therapy [10]. Our patient, however, had undergone seven prior dilatations with recurrent obstruction, ultimately necessitating a surgical referral. This potentially reflects a more refractory and fibrotic stricture than is typically described. Additionally, the breadth of multidisciplinary involvement, critical care, neurology, cardiology, gastroenterology, and dietetics was notable compared with prior reports that primarily emphasise gastroenterological management.

In summary, this case adds to the sparse Western literature describing pyloric obstruction as a cause of acute flaccid paralysis. The differences identified multisystem presentation, persistence of weakness despite repletion, cardiac mimicry, and refractory obstruction highlight the variable ways in which this entity may manifest. Greater awareness of such metabolic mimics of GBS and ACS is essential, as timely recognition and collaborative management can prevent misdiagnosis, unnecessary interventions, and poor outcomes.

## Conclusions

Pyloric stricture can present with chronic vomiting, malnutrition, and severe electrolyte derangements. In this case, profound hypokalaemia led to functional weakness and T2MI, initially mimicking neurological and cardiac emergencies. Recognition of the underlying gastrointestinal obstruction was key to explaining the patient’s multisystem presentation.

This report underscores the importance of considering pyloric stenosis as a driver of metabolic and nutritional crises in adults with recurrent vomiting. Early multidisciplinary input, including gastroenterology, critical care, dietetics, and surgery, was essential in stabilising the patient and planning definitive management. Timely recognition and treatment of both the metabolic complications and the structural cause can significantly improve outcomes.
